# Self-organizing neural integration of pose-motion features for human action recognition

**DOI:** 10.3389/fnbot.2015.00003

**Published:** 2015-06-09

**Authors:** German I. Parisi, Cornelius Weber, Stefan Wermter

**Affiliations:** Department of Informatics, Knowledge Technology Institute, University of HamburgHamburg, Germany

**Keywords:** action recognition, visual processing, depth information, neural networks, self-organizing learning, robot perception

## Abstract

The visual recognition of complex, articulated human movements is fundamental for a wide range of artificial systems oriented toward human-robot communication, action classification, and action-driven perception. These challenging tasks may generally involve the processing of a huge amount of visual information and learning-based mechanisms for generalizing a set of training actions and classifying new samples. To operate in natural environments, a crucial property is the efficient and robust recognition of actions, also under noisy conditions caused by, for instance, systematic sensor errors and temporarily occluded persons. Studies of the mammalian visual system and its outperforming ability to process biological motion information suggest separate neural pathways for the distinct processing of pose and motion features at multiple levels and the subsequent integration of these visual cues for action perception. We present a neurobiologically-motivated approach to achieve noise-tolerant action recognition in real time. Our model consists of self-organizing Growing When Required (GWR) networks that obtain progressively generalized representations of sensory inputs and learn inherent spatio-temporal dependencies. During the training, the GWR networks dynamically change their topological structure to better match the input space. We first extract pose and motion features from video sequences and then cluster actions in terms of prototypical pose-motion trajectories. Multi-cue trajectories from matching action frames are subsequently combined to provide action dynamics in the joint feature space. Reported experiments show that our approach outperforms previous results on a dataset of full-body actions captured with a depth sensor, and ranks among the best results for a public benchmark of domestic daily actions.

## 1. Introduction

For humans and other mammals, the recognition of others' actions represents a crucial ability underlying social interaction and perceptual decision-making. Similarly, the visual recognition of complex movements may be fundamental for artificial systems to enable natural human-robot interaction (HRI) and action-driven social perception (Layher et al., 2012). The robust classification of full-body, articulated actions represents a key component of assistive robots aiming to provide reliable recognition of user behavior and remains an enticing milestone for artificial systems embedded in socially-aware agents (Kachouie et al., 2014). When operating in complex environments, algorithms for the visual recognition of action classes face a trade-off between satisfactory accuracy and minimal recognition latency (Ellis et al., 2013). There exists a vast set of challenges to be addressed regarding the efficient processing of raw visual information and the generalization of actions for effective inter-class discrimination, while neglecting subtle intra-class differences. Moreover, an enduring bottleneck for vision-based approaches regards the segmentation of human shape and motion from 2D image sequences, often constrained in terms of computational efficiency and robustness to illumination changes (Weinland et al., 2011).

In the last half decade, the use of low-cost depth sensing devices such as the Microsoft Kinect and ASUS Xtion has led to a great number of vision-based applications using depth information instead of, or in combination with, brightness and color information (for a review see Han et al., 2013). This sensor technology provides depth measurements used to obtain reliable estimations of 3D human motion in cluttered environments, including a set of body joints in real-world coordinates and limb orientations. Despite recent research efforts combining 3D skeleton models with machine learning and neural network approaches, the question remains open on how to better process extracted body features for effectively learning the complex dynamics of actions in real-world scenarios. For instance, in such scenarios the correct classification of actions may be hindered by noisy and missing body joints caused by systematic sensor errors or temporary occluded body parts (Parisi and Wermter, 2013). Nevertheless, a robust, noise-tolerant system should also operate under such adverse conditions. A promising scheme to tackle this demanding task is the implementation of computational models built upon evidence from the biological visual system. This scheme is supported by the fact that human observers are capable of carrying out action discrimination effortlessly (Blake and Shiffrar, 2007), outperforming artificial systems. In particular, neural mechanisms underlying action recognition have been broadly studied in the literature (Perrett et al., 1982; Giese and Poggio, 2003), thereby encompassing multidisciplinary research to shed light on perceptual representations and neural pathways responsible for triggering robust action perception in humans and non-human primates. Simplified models of brain areas processing visual cues have been adopted as a stepping stone to numerous artificial systems dealing with the detection and classification of articulated, complex motion such as human actions (Giese and Poggio, 2003; Layher et al., 2012).

In this work, we present a learning architecture for the recognition of actions based on the following three assumptions consistent with neurobiological evidence from the mammalian visual system: (1) Complex motion is analyzed in parallel by two separated pathways and subsequently integrated to provide a joint percept (Perrett et al., 1982; Vangeneugden et al., 2009); (2) Both channels contain hierarchies to extrapolate shape and optic-flow features with increasing complexity (Giese and Poggio, 2003), from low- to high-level representations of the visual stimuli; (3) Input-driven self-organization is crucial for the cortex to tune the neurons according to the distribution of the inputs (von der Malsburg, 1973; Kohonen, 1993; Miikkulainen et al., 2005). Under these assumptions, we carry out action learning and classification through a two-pathway hierarchy of growing self-organizing networks that cluster separately pose and motion samples. During the training, Growing When Required networks (Marsland et al., 2002) dynamically change their topological structure through competitive Hebbian learning (Martinetz, 1993) to incrementally match the input space. The learning process is built upon input-driven synaptic plasticity (Pascual-Leone et al., 2011) and habituation (Thompson and Spencer, 1966). Clustered neuronal activation trajectories from the parallel pathways are subsequently integrated to generate prototype neurons representing action dynamics in the joint pose-motion domain, resembling the neural integration of multi-cue action features in the visual cortex (Beauchamp et al., 2003).

In previous research we explored the use of hierarchical self-organization for integrating pose-motion cues using Growing Neural Gas (GNG) learning (Parisi et al., 2014a,c). The unsupervised learning algorithm was extended with two labeling functions for classification purposes. In this work, we use GWR networks that can create new neurons whenever the activity of the best neuron matching the input is not sufficiently high, leading to a more efficient convergence with respect to GNG networks that use a fixed insertion interval. In the previous model, an extra network was used to automatically detect outliers in the training and test set. However, the removal of noisy cues via an additional specialized network lacks neurobiological support and adds complexity to the model. With the use of an extended GWR learning mechanism, we will show that this process can be embedded naturally into the self-organizing hierarchy for the clustering of action cues and allows to remove noisy samples also during live classification.

The rest of this paper is structured as follows. In Section 2, we introduce biological evidence and models for neural integration of multiple visual cues and present an overview of state-of-the-art learning approaches for human action recognition using depth information. In Section 3, we present our hierarchical self-organizing architecture and the learning GWR algorithm extended for the classification of new action samples. In Section 4, we provide experimental results along with an evaluation of our classification algorithm on a dataset of 10 full-body actions (Parisi et al., 2014c) and a benchmark of domestic actions CAD-60 (Sung et al., 2012). We conclude in Section 5 with a discussion on the neurobiological aspects of action recognition and foundations underlying our approach, as well as future work directions for recognition systems embedded in assistive robots and HRI scenarios.

## 2. Recognition of human actions

### 2.1. Processing of pose and motion in biology

In humans, the skill to recognize human movements arises in early life. The ability of neonates to imitate manual gestures suggests that the recognition of complex motion may depend on innate neural mechanisms (Meltzoff and Moore, 1977). Studies on preferential looking with 4-month-old infants evidence a preference for staring at human motion sequences for a longer duration than sequences with random motion (Bertenthal and Pinto, 1993). Behavioral testing has shown that young children aged three to five steadily enhance their skills to identify human and non-human biological motion portrayed as animations of point-light tokens and reach adult performance by the age of five (Pavlova et al., 2001). Psychophysiological experiments on the discrimination of actions reported a remarkable efficiency of adult human observers to temporally integrate biological motion also under noisy conditions, i.e., impoverished and potentially ambiguous visual stimuli (Neri et al., 1998). However, action perception has been shown to be disrupted by perturbations in the temporal relations of both biological and artificial motion morphs (Bertenthal and Pinto, 1993; Jastorff et al., 2006), suggesting that the recognition of complex motion is highly selective to temporal order (Giese and Poggio, 2003). Additionally, it has been found that learning plays an important role in complex motion discrimination. Studies showed that the recognition speed and accuracy of humans have improved after a number of training sessions, not only for biologically relevant motion but also for artificial motion patterns underlying a skeleton structure (Jastorff et al., 2006; Hiris, 2007).

Early neurophysiological studies have identified a specialized area for the visual coding of complex, articulated motion in the non-human mammalian brain (Perrett et al., 1982). An extensive number of supplementary studies has shown that the mammalian visual system processes biological motion in two separate neural pathways (Giese and Poggio, 2003). The ventral pathway recognizes sequences of snapshots of body postures, while the dorsal pathway recognizes movements in terms of optic-flow patterns. Both pathways comprise hierarchies that extrapolate visual features with increasing complexity of representation. Although there has been a long-standing debate on which visual cue was predominant to action coding, i.e., either posture (Lange et al., 2006) or motion (Troje, 2002), additional studies have found neurons in the macaque superior temporal sulcus (STS) that are sensitive to both motion and posture for representing similarities among actions, thus suggesting contributions from converging cues received from the ventral and dorsal pathways (Oram and Perrett, 1996). On the basis of additional studies showing that neurons in the human STS activate by body articulation (Beauchamp et al., 2003), there is a consensus that posture and motion together play a key role in biological motion perception (Garcia and Grossman, 2008; Thirkettle et al., 2009). These findings have served to the development of architectures using learned prototype patterns to recognize actions, consistent with the idea that STS neurons integrate both body pose and motion (Vangeneugden et al., 2009). Computational feed-forward models have been developed to learn action dynamics processed as pose-motion cue patterns with recognition selective to temporal order (Giese and Poggio, 2003; Layher et al., 2012; Tan et al., 2013).

### 2.2. Machine learning and depth-based recognition

Other methodologies without biological foundations have also been successfully applied to action recognition. Machine learning techniques processing multi-cue features from natural images have shown motivating results for classifying a set of training actions. For instance, Xu et al. (2012) presented a system for action recognition using dynamic poses by coupling local motion information with pose in terms of skeletal joint points. They generated a codebook of dynamic poses from two RGB action benchmarks (KTH and UCF-Sports), and then classified these features with an Intersection Kernel Support Vector Machine. Jiang et al. (2012) explored a prototype-based approach using pose-motion features in combination with tree-based prototype matching via hierarchical clustering and look-up table indexing for classification. They evaluated the algorithm on the Weizmann, KTH, UCF Sports, and CMU action benchmarks. To be noted is that although these two approaches use pose-motion cues to enhance classification accuracy with respect to traditional single-cue approaches, they do not take into account an integration function that learns order-selective prototypes of joint pose-motion representations of action segments from training sequences. Furthermore, these classification algorithms can be susceptible to noise or missing observations which may occur during live recognition.

Learning systems using depth information from low-cost sensors are increasingly popular in the research community encouraged by the combination of computational efficiency and robustness to light changes in indoor environments. In recent years, a large number of applications using 3D motion information has been proposed for human activity recognition such as classification of full-body actions (Faria et al., 2014; Shan and Akella, 2014; Parisi et al., 2014c), fall detection (Rougier et al., 2011; Mastorakis and Makris, 2012; Parisi and Wermter, 2013), and recognition of hand gestures (Suarez and Murphy, 2012; Parisi et al., 2014a,b; Yanik et al., 2014). A vast number of depth-based methods has used a 3D human skeleton model to extract relevant action features for the subsequent use of a classification algorithm. For instance, Sung et al. (2012) combined the skeleton model with Histogram of Oriented Gradient features and then used a hierarchical maximum entropy Markov model to classify 12 different actions. The learning model used a Gaussian mixture model to cluster and segment the original training data into activities. Using the same action benchmark for the evaluation, Shan and Akella (2014) used action templates computed from 3D body poses to train multiple classifiers: Hidden Markov Model, Random Forests, K-Nearest Neighbor, and Support Vector Machine (SVM). Faria et al. (2014) used a dynamic Bayesian Mixture Model designed to combine multiple classifier likelihoods and compute probabilistic body motion. Zhu et al. (2014) evaluated a set of spatio-temporal interest point features from raw depth map images to classify actions with a SVM. Experiments were conducted also using interest points in combination with skeleton joint positions and color information, obtaining better results. However, the authors also showed that noisy depth data and cluttered background have a great impact on the detection of interest points, and that actions without much motion are not well recognized. The performance of the above mentioned approaches on the CAD-60 benchmark is listed in Table 2 (Section 4).

## 3. Self-organizing neural architecture

### 3.1. Overview

**Figure 1:**
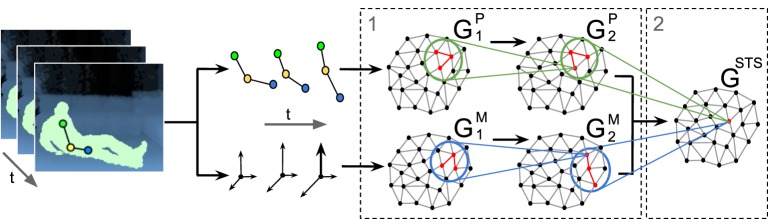
**GWR-based architecture for the processing of pose-motion samples.** (1) Hierarchical processing of pose-motion features in parallel. (2) Integration of neuron trajectories in the joint pose-motion feature space.

Our architecture consists of a two-stream hierarchy of Growing When Required (GWR) networks that processes extracted pose and motion features in parallel and subsequently integrates clustered neuronal activation trajectories from both streams. This latter network resembles the response of STS model neurons encoding sequence-selective prototypes of action segments in the joint pose-motion domain. An overall overview of the architecture is depicted in Figure 1. To enable the classification of new action samples, we assign labels to STS prototype neurons by extending the GWR algorithm with two offline labeling functions. We process pose and motion cues under the assumption that action recognition is selective for temporal order (Bertenthal and Pinto, 1993; Giese and Poggio, 2003). Therefore, positive recognition of action segments occurs only when neurons along the hierarchy are activated in the correct order of learned movement sequences.

### 3.2. Input-driven self-organization

The visual system is composed of topographically arranged structures that organize according to environmental stimuli (Hubel and Wiesel, 1962; von der Malsburg, 1973; Hubel and Wiesel, 1977; Miikkulainen et al., 2005). This neural foundation, referred to as input-driven self-organization, has been shown to shape the connections in the visual cortex according to the distribution of the inputs. From a computational perspective, self-organization is an unsupervised mechanism that allows to learn representations of the input by adaptively obtaining a projection of the feature space (Kohonen, 1993).

Similar to biological mechanisms for synaptic plasticity in areas of the visual cortex, computational models may exhibit learning capabilities through the development of lateral connections between nodes governed by the principle formulated by Hebb (1949), in which nodes that are concurrently activated increase their synaptic strength. The simplest formulation of the Hebbian rule is as follows:

(1) $$\Delta C_{ij}\propto y_{i}\cdot y_{j}\;,$$

denoting that the change of the connection strength *C*_*ij*_ is proportional to the presynaptic activity *w*_*i*_ and the postsynaptic activity *w*_*j*_. Self-organizing networks introduce competition among nodes such that connectivity patterns become structured by activating only the neuron with the highest similarity to the input, and thus progressively reflecting topological properties of the input distribution. This mechanism, referred to as competitive Hebbian learning (CHL) (Martinetz, 1993), creates (or strengthens) the connection between the winner and the second-nearest neuron during the learning phase. The best matching neuron *w*_*b*_ is computed using a distance function (usually an Euclidean metric) so that, for an input signal ξ and the set of *g* neurons, the following condition holds:

(2) $$\|\xi -w_{b}\|\;<\;\|\xi -w_{g}\|\;.$$

Neural network approaches inspired by biological self-organization such as self-organizing maps (SOM) (Kohonen, 1995) and neural gas (NG) (Martinetz and Schluten, 1991) have shown to be a simplified, yet plausible model for clustering human motion patterns in terms of multi-dimensional flow vectors (Parisi and Wermter, 2013). The advantage of these networks lies in their ability to learn the topological relations of the input space without supervision. The process is carried out with the use of the vector quantization technique in which a layer of competitive neurons will represent prototype vectors that encode a submanifold of the input space with a small representation error. In the SOM, each neuron of the competitive layer is connected to adjacent neurons by a neighbourhood relation that defines the structure of the map. Growing self-organizing networks represent one approach to address the limitations of the SOM and NG in which the number of neurons must be fixed beforehand and cannot be changed over time. The Growing Neural Gas (GNG) proposed by Fritzke (1995) has the ability to add new neurons to an initially small network by evaluating local statistical measures on the basis of previous adaptations, and to create and remove connections between existing neurons. The network topology is generated incrementally through CHL, i.e., for each input vector, a connection is generated between the neuron that best matches the input and the second-best matching neuron. New neurons are added when the number of learning iterations performed is a multiple of a predefined constant. This allows us to use the GNG algorithm also in on-line learning scenarios. However, the fixed neuron insertion interval has the limitation that the network grows at the same rate no matter how the input distribution is changing.

### 3.3. A growing when required network

The Growing When Required (GWR) algorithm by Marsland et al. (2002) decides when to add new neurons by evaluating the activity of that neuron that best matches the current input. The GWR network is composed of a set of neurons, from now on referred to as *nodes*, with their associated weight vectors, and the edges that link the nodes. Similar to the GNG, the GWR network topology is generated through CHL (Martinetz, 1993). However, in the GWR nodes can be created at any time depending on the input. The network starts with a set of two nodes randomly initialized from within the training data. At each time step, both the nodes and the edges can be created and removed. The node activity is computed as a function of the distance between the input and the node weights. Furthermore, each node is equipped with a mechanism to measure how often the node has fired to foster the training of existing nodes over creating unnecessary ones. Edge connections have an associated age that will be used to remove old connections. At each iteration, nodes without connections are deleted.

The learning is carried out by adapting the position of the best-matching neurons and its neighbors. This learning mechanism takes into account the number of times that a node has fired so that nodes that have fired frequently are trained less. In animals, this decreasing response of neurons to a stimulus that has been frequently presented is known as habituation (Kohonen, 1993). Stanley (1976) proposed a differential equation as a simplified model of how the efficacy of an habituating synapse reduces over time:

(3) $$\tau \frac{dh_{s}(t)}{dt}=\alpha [h_{0}-h(t)]-S(t)\;,$$

where *h*_*s*_(*t*) is the size of the firing rate for node *s*, *h*_0_ is a the resting value, *S*(*t*) is the stimulus strength, and τ, α are constants that control the behavior of the curve. The solution to Equation (3) can therefore provide a habituation counter *h*(*t*) of how frequently a node *s* has fired:

(4) $$h(t)=h_{0}-\frac{S(t)}{\alpha }\cdot (1-e^{\left(-\alpha_{t}/\tau \right)})\;.$$

The GWR algorithm will iterate over the training set until a given stop criterion is met, e.g., a maximum network size (number of nodes) or a maximum number of iterations.

Let *A* be the set of nodes, *C* ⊂ *A* × *A* the set of connections between them, *P*(ξ) the distribution of the input ξ of dimension *k*, and *w*_*n*_ the *k*-dimensional weight vector of a node *n* ∈ *A*. The GWR training algorithm is given by Algorithm 1 (Marsland et al., 2002).

**Algorithm 1:** Growing When Required

1:	Start with a set *A* consisting of two map nodes, *n*_1_ and *n*_2_, at random positions.
2:	Initialize an empty set of connections *C* = ∅.
3:	At each iteration, generate an input sample ξ according to the input distribution *P*(ξ).
4:	For each node *i* calculate the distance from the input ||ξ − *w*_*i*_||.
5:	Select the best matching node and the second-best matching node such that: *s* = arg min_*n* ∈ *A*_||ξ − *w*_*n*_||, *t* = arg min_*n* ∈ *A*/{*s*}_ ||ξ − *w*_*n*_||.
6:	Create a connection *C* = *C* ∪ {(*s*, *t*)} if it does not exist and set its age to 0.
7:	Calculate the activity of the best matching unit: *a* = *exp*(−||ξ − *w*_*s*_||).
8:	If *a* < activity threshold *a*_*T*_ and firing counter < firing threshold *f*_*T*_ then: Add a new node between *s* and *t*: *A* = *A* ∪ {(*r*)} Create the weight vector: *w*_*r*_ = 0.5 · (*w*_*s*_ + ξ) Create edges and remove old edge: *C* = *C* ∪ {(*r*, *s*), (*r*, *t*)} and *C* = *C*/{(*s*, *t*)}.
9:	Else, i.e., no new node is added, adapt the positions of the winning node and its neighbours *i*: Δ*w*_*s*_ = ϵ_*b*_ · *h*_*s*_ · (ξ − *w*_*s*_) Δ*w*_*i*_ = ϵ_*n*_ · *h*_*i*_ · (ξ − *w*_*i*_) where 0 < ϵ_*n*_ < ϵ_*b*_ < 1 and *h*_*s*_ is the value of the firing counter for node *s*.
10:	Increment the age of all edges connected to *s*: *age*_(*s*,*i*)_ = *age*_(*s,i*)_ + 1.
11:	Reduce the firing counters according to Equation (2): $h_{s}(t)=h_{0}-\frac{S(t)}{\alpha_{b}}\cdot (1-exp(-\alpha_{b}t/\tau_{b}))$ $h_{i}(t)=h_{0}-\frac{S(t)}{\alpha_{n}}\cdot (1-exp(-\alpha_{n}t/\tau_{n}))$.
12:	Remove all edges with ages larger than *a*_*max*_ and remove nodes without edges.
13:	If the stop criterion is not met, go to step 3.

The values for the reported experiments with stationary datasets were: insertion thresholds *a*_*T*_ = 0.95, learning rates ϵ_*b*_ = 0.2 and ϵ_*n*_ = 0.006, maximum age threshold *a*_*max*_ = 50, firing counter *h*_0_ = 1, and habituation parameters α_*b*_ = 0.95, α_*n*_ = 0.95, and τ_*b*_ = 3.33.

### 3.4. Noise detection

The presence of noise in the sense of outliers in the training set has been shown to have a negative influence on the formation of faithful topological representations using SOMs (Parisi and Wermter, 2013), whereas such an issue is partially addressed by incremental networks. For instance, incremental networks such as GNG and GWR are equipped with a mechanism to remove rarely activated nodes and connections that may represent noisy input (Algorithm 1, step 12). In contrast to GNG, however, the learning strategy of the GWR shows a quick response to changes in the distribution of the input by creating new neurons to match it. The insertion threshold *a*_*T*_ modulates the number of neurons that will be added, e.g., for high values of *a*_*T*_ more nodes will be created (Algorithm 1, step 8). However, the network is also equipped with a mechanism to avoid slight input fluctuations to perturb the learning convergence and the creation of unnecessary nodes. The GWR takes into account the number of times that a neurons has been activated, so that neurons that have been activated more times, are trained less. Therefore, an additional threshold modulates the firing counter of neurons so that during the learning process less trained neurons are updated, whereas new neurons are created only when existing neurons do not sufficiently represent the input. A number of experiments have shown that the GWR is well-suited for novelty detection (Marsland et al., 2002), which involve the identification of inputs that do not fit the learned model.

**Figure 2:**
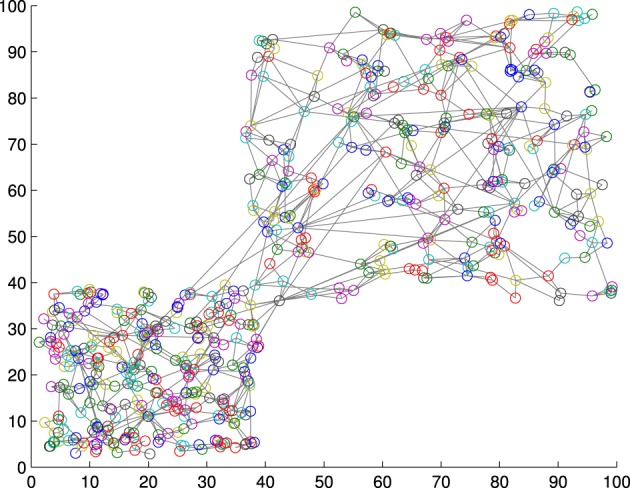
**A GWR network trained with a normally distributed training set of 1000 samples resulting in 556 nodes and 1145 connections**.

**Figure 3:**
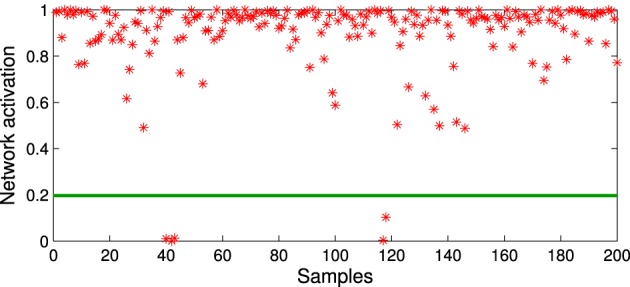
**Activation values for the network trained in Figure 2 with a test set of 200 samples containing noise.** Noisy samples line under novelty threshold *a*_*new*_ = 0.1969 (green line).

In line with this mechanism, we use the activation function (Algorithm 1, step 7) to detect noisy input after the training phase. The activation function will be equal to 1 in response to input that perfectly matches the model, i.e., minimum distance between the weights of the neuron and the input, and will decrease exponentially for input with a higher distance. If the response of the network to the novel input is below a given novel activation threshold *a*_*new*_, then the novel input can be considered noisy in the sense that it is not represented by well-trained prototype neurons, and thus discarded. The threshold value *a*_*new*_ can be empirically selected by taking into account the response distribution of the trained network with respect to the training set. For each novel input *x*_*new*_, we compute:

(5) $$exp\left\{-\sqrt{\sum_{j\;=\;1}^{k}(x_{new,j}-s(x_{new,j}){)}^{2}}\right\}<\bar{A}-\gamma \cdot \sigma (A)\;,$$

where Ā and σ(*A*) are respectively the mean and the standard deviation of the set of activations *A* is obtained from the training set, and γ is a constant value that modulates the influence of fluctuations in the activation distribution. Figure 2 shows a GWR network trained with 100 input vectors with two normally distributed clusters. Over its 500 iterations, the network created 556 neurons and 1145 connections (*a*_*T*_ = 0.95, γ = 4). The activation values for a test set of 200 samples (also normally distributed) containing artificially introduced noise are shown in Figure 3. It is observable how noisy samples lie below the computed activation threshold *a*_*new*_ = 0.1969 (Equation 5) and can, therefore, be discarded. We use this noise detection procedure to all the networks in our architecture with the aim to attenuate noise in the training data and prevent the forced classification of input that are not represented by the trained model.

### 3.5. Hierarchical learning and integration

The motivation underlying our hierarchical learning is to use trajectories of neuron activations from one network as input for the training for a subsequent network. This mechanism allows to obtain progressively specialized neurons coding inherent spatio-temporal dependencies of the input, consistent with the assumption that the recognition must be selective for temporal order.

Hierarchical training is carried out as follows. We first train a network *G* with a training set *T*. After the training is completed, the subsequent network G^*^ will be trained with a new set T^*^ that is obtained computing trajectories of best-matching neurons from *G* for samples of *T*. For each *k*-dimensional sample *x* ∈ *T*, we compute the best-matching neuron as

(6) $$s(x)=\underset{\;n\;\in \;A}{\arg\min}\sqrt{\sum_{j\;=\;1}^{k}{\left(x_{j}-w_{n,j}\right)}^{2}}\;,$$

from which we can compute a trajectory of prototype neurons of length *q*:

(7) $$\omega (x_{i})=\{s(x_{i}),s(x_{i\;-\;1}),\ldots ,s(x_{i\;-\;q+1}),i\in [q..m]\}\;,$$

where *m* is the number of samples of *T*. The next step is to compute the training set T^*^ by concatenating the *m* − *q* trajectories of neuron activations over *T* with a temporal sliding window scheme, in our specific case using activation trajectories with 3 neurons (*q* = 3) for all the stages. The training of G^*^ will then produce a network with neurons encoding temporally-ordered prototype sequences from consecutive samples of *T*.

At the first stage of our hierarchy, each stream is composed of two GWR networks to process pose and motion features separately. We therefore compute two distinct datasets with sequentially-ordered pose and motion features, denoted as *P* and *M* respectively. Since *P* and *M* are processed by different network hierarchies, they can differ in dimensionality. Following the notation introduced in Figure 1, we train the networks *G*^*P*^_1_ and *G*^*M*^_1_ with samples from *P* and *M* respectively. After this step, we train *G*^*P*^_2_ and *G*^*M*^_2_ with the training sets of concatenated trajectories of best-matching neurons (Equation 7).

The STS stage consists of the integration of prototype activation trajectories from both streams by training the network *G*^*STS*^ with two-cue trajectory samples. For this purpose, we compute a new dataset *T*^*STS*^ by merging best-matching trajectories from *G*^*P*^_2_ and *G*^*M*^_2_ into a set of trajectory pairs ψ_*u*_ as follows:

(8) $$\begin{array}{l} \psi_{u}\text{​​​​​​}=\text{ ​​​​}\{s(\omega (x_{i})),\ldots ,s(\omega (x_{i\;-\;q-1})),s(\omega (y_{i})),\ldots ,s(\omega (y_{i\;-\;q-1})),\\ \;x_{i}\in P,y_{i}\in M,u\in [q..m-q]\}\;.\text{​​​​} \end{array}$$

After the training of *G*^*STS*^ is completed, each neuron will encode a sequence-selective prototype action segment, thereby integrating changes in the configuration of a person's body pose over time.

### 3.6. Classification

At recognition time, our goal is to process and classify unseen action sequences to match one of the training actions. For this purpose, we extend the unsupervised GWR-based learning with two labeling functions: one for the training phase and one for returning the label of unseen samples.

Let *L* be the set of *j* action classes that we want to recognize, for instance “walk” and “fall down.” We then assume that each action δ_*j*_ will be therefore composed of a set of labeled, sequentially-ordered feature vectors:

(9) $$\delta_{j}=\{(F_{i},l_{j}):i\in [1..v],l_{j}\in L\}\;,$$

where *l*_*j*_ is the action label and *v* is the number of feature vectors *f* ∈ *F*_*i*_ for the action class δ_*j*_. Sample labels are not used during the first stage of the learning process. The learning process is carried out without supervision following Algorithm 1. In addition, each neuron of the STS network will be assigned an action label during the training phase. We train the *G*^*STS*^ network with the labeled training pairs (ψ_*u*_, *l*_*j*_) and define a labeling function *l*: *N* → *L* for the training phase, where *N* is the set of nodes. We adopted the labeling technique that has shown to achieve best classification accuracy among other labeling strategies for GNG-based learning discussed by Beyer and Cimiano (2011). According to a minimal-distance strategy, the sample ψ_*u*_ will adopt the label *l*_*j*_ of the closest ψ:

(10) $$l(\psi_{k})=l_{j}=l(\underset{\;\psi \in \Psi }{\arg\min}\|\psi_{i}-\psi {\|}^{2})\;.$$

This labeling procedure works in an offline mode since we assume that all training samples and labels are available a priori. This mechanism requires the extension of the standard GWR algorithm for assigning a training label to the best-matching neuron of the current input (Algorithm 1, step 5).

For the classification task, we define a recognition function ψ: Ψ → *L* on the basis of a single-linkage strategy (Beyer and Cimiano, 2011) in which a new sample Ψ_*new*_ is labeled with *l*_*j*_ associated to the neuron *n* that minimizes the distance to the new sample:

(11) $$\varphi (\psi_{new})=\underset{\;l_{j}}{\arg\min}(\arg\underset{n\;\in \;N(l_{j})}{\min}\|n-\psi_{new}{\|}^{2})\;.$$

The hierarchical flow is composed of 3 networks with each subsequent network neuron encoding a window of 3 samples from the previous one. Therefore, this classification algorithm returns the first action label *l*_*new*_ after 9 new samples $\hat{f}$ ∈ *F*. Then, applying the temporal sliding window scheme, we get a new action label for each new sample. For instance, operating at 15 frames per second, we would get the first action label after 9/15 = 0.6 s.

## 4. Results

We evaluated our approach both on our action dataset (Parisi et al., 2014c) and the public action benchmark CAD-60 (Sung et al., 2012). We now provide details on feature extraction, learning parameters for the GWR-based training and recognition, and a comparative evaluation.

### 4.1. Action features

#### 4.1.1. Full-body actions

Our action dataset is composed of 10 full-body actions performed by 13 student participants with a normal physical condition. Participants were naive as to the purpose of the experiment and they had not been explained how to perform the actions in order to avoid biased execution. They were recorded individually and gave written consent to participate in the study. We monitored the participants in a home-like environment with a Kinect sensor installed 130 m above the ground. Depth maps were sampled with a VGA resolution of 640 × 480, an operation range from 0.8 to 3.5 meters and a constant frame rate of 30 Hz. The dataset contained periodic and goal-oriented actions:

- Periodic: Standing, walking, jogging, sitting, lying down, crawling (10 min each);
- Goal-oriented: Pick up object, jump, fall down, stand up (60 repetitions each).

**Figure 4:**
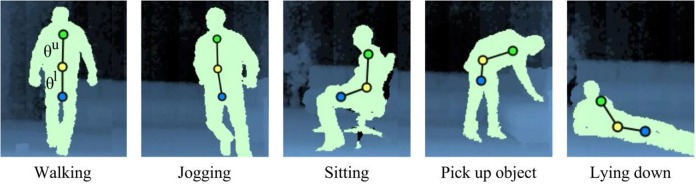
**Representation of full-body movements from our action dataset.** We estimate three centroids *C*_1_ (green), *C*_2_ (yellow) and *C*_3_ (blue) for upper, middle and lower body respectively. The segment slopes θ^*u*^ and θ^*l*^ describe the posture in terms of the overall orientation of the upper and lower body.

From the raw depth map sequences, 3D body joints were estimated on the basis of the tracking skeleton model provided by OpenNI^1^. We represented whole-body actions in terms of three body centroids (Figure 4): *C*_1_ for upper body with respect to the shoulders and the torso; *C*_2_ for middle body with respect to the torso and the hips; and *C*_3_ for lower body with respect to the hips and the knees. Each centroid is computed as a point sequence of real-world coordinates *C* = (*x*, *y*, *z*). To attenuate sensor noise, we used the median value of the last 3 estimated points. We then estimated upper and lower orientations θ^*u*^ and θ^*l*^ given by the slope angles of the line segments {*C*_1_, *C*_2_} and {*C*_2_, *C*_3_} respectively. As shown in Figure 4, the values θ^*u*^ and θ^*l*^ describe the overall body pose according to the orientation of the torso and the legs, which allows to capture significant pose configurations in actions such as walking, sitting, picking up and lying down. We computed the body velocity *S*_*i*_ as the difference in pixels of the centroid *C*_1_ between two consecutive frames *i* and *i* − 1. The upper centroid was selected based on the motivation that the orientation of the torso is the most characteristic reference during the execution of a full-body action (Papadopoulos et al., 2014). We then computed horizontal speed *h*_*i*_ and vertical speed *v*_*i*_ (Parisi and Wermter, 2013). For each action frame *i*, we computed the following pose-motion vector:

(12) $$F_{i}=(\theta_{i}^{u},\theta_{i}^{l},h_{i},v_{i})\;.$$

Thus, each action *A*_*j*_ will be composed of a set of sequentially ordered pose-motion vectors such that:

(13) $$A_{j}:=\{(F_{i},l_{j}):i\in [1..n],l_{j}\in L\}\;,$$

where *l*_*j*_ is the action label, *L* is the set of class labels, and *n* is the number of training vectors for the action *j*. Action labels were manually annotated for video sequences containing one action. We divided the data equally into training and test set, i.e., 30 sequences of 10 s for each periodic action and 30 repetitions for each goal-oriented action. Both the training and test sets contained data from all participants. For a fair comparison with previous results (Parisi et al., 2014c), we adopted similar feature extraction and evaluation schemes.

#### 4.1.2. CAD60

**Figure 5:**
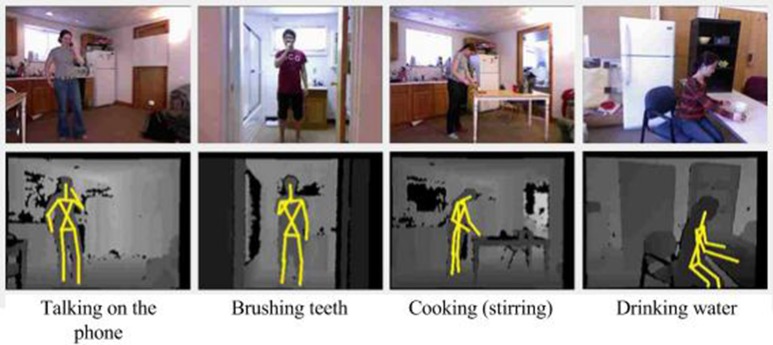
**Daily actions from the CAD-60 dataset (RGB and depth images with skeleton)**.

The Cornell activity dataset CAD-60 (Sung et al., 2012) is composed of 60 RGB-D videos of four subjects (two males, two females, one left-handed) performing 12 activities: *rinsing mouth, brushing teeth, wearing contact lens, talking on the phone, drinking water, opening pill container, cooking (chopping), cooking (stirring), talking on couch, relaxing on couch, writing on whiteboard, working on computer*. The activities were performed in 5 different environments: office, kitchen, bedroom, bathroom, and living room. The videos were collected with a Kinect sensor with distance ranges from 1.2 to 3.5 m and a depth resolution of 640×480 at 15 frames per second. The dataset provides raw depth maps and RGB images, and skeleton data. An example of the actions and the resulting skeletons is shown in Figure 5. The dataset provides skeleton data composed of 15 extracted joints for the following body parts: *head, neck, torso, shoulders, elbows, hands, hips, knees*, and *feet*.

For our approach, we used the set of 3D positions without the *feet*, leading to 13 joints (i.e., 39 input dimensions). Instead of using world coordinates, we encoded the joint positions using the center of the hips as frame of reference to obtain translation invariance. We then computed joint motion as the difference of two consecutive frames for each pose transition. We added a mirrored version of all action samples to obtain invariance to actions performed with either the right or the left hand.

### 4.2. Training

We now report the GWR parameters for the training sessions. We set the following values: insertion thresholds *a*_*T*_ = 0.90, learning rates ϵ_*b*_ = 0.3, and ϵ_*n*_ = 0.006, maximum age *a*_*max*_ = 50, firing counter parameters *h*_0_ = 1, τ_*b*_ = 0.3, τ_*n*_ = 0.1. Each network stopped training after a 500 epochs over the whole dataset. These parameters were empirically found to let the model learn spatio-temporal dependencies with the best accuracy in terms of classification labels returned by the last network *G*^*STS*^. For a single network, the number of neurons converged already after 100 epochs, and weight vectors of neurons showed little modification after 400 epochs. If we consider the 2 networks per stream in the first stage of the hierarchy and the integration network in the second stage (Figure 1), it took overall 1500 epochs to obtained a trained neuron in the *G*^*STS*^ network.

In Table 1, we show the resulting properties of the networks along the hierarchy after the training sessions on the two datasets. In both cases, it can be observed that the number of nodes (*N*) and connections (*C*) is lower for higher levels of the hierarchy. The lower numbers indicate that in the STS level neurons encode more complex spatio-temporal dependencies with respect to the first level (in which only uni-cue spatial relations are considered), but with a smaller number of specialized neurons. To be noticed is that the number of neurons did not depend on the dimensionality of the input, but rather on the distribution of the data. From Table 1 it can also be seen that the activation threshold (*a*) increases toward higher levels of the hierarchy. In the first level, the activation function yielded larger fluctuations due to outliers and input data that were rarely presented to the network during the training. Conversely, activations of training samples matching the model get higher as neurons specialize. These results indicate that noise from the training data was not propagated along the hierarchy, but rather detected and discarded, which leads to a larger *a*-value.

**Table 1:** **Training results on the two datasets—For each trained network along the hierarchy, the table shows the resulting number of nodes (*****N*****) and connections (*****C*****), and the activation threshold (*****a*****)**.

Full-body actions	*G*^*P*^_1_	*N* = 225 *C* = 435 *a* = 0.1865	*G*^*P*^_2_	*N* = 183 *C* = 338 *a* = 0.1934	*G*^*STS*^	*N* = 118 *C* = 378 *a* = 0.2932
	*G*^*M*^_1_	*N* = 254 *C* = 551 *a* = 0.1732	*G*^*M*^_2_	*N* = 192 *C* = 353 *a* = 0.1910		
CAD-60	*G*^*P*^_1_	*N* = 289 *C* = 403 *a* = 0.1778	*G*^*P*^_2_	*N* = 214 *C* = 445 *a* = 0.1898	*G*^*STS*^	*N* = 137 *C* = 309 *a* = 0.2831
	*G*^*M*^_1_	*N* = 302 *C* = 542 *a* = 0.1698	*G*^*M*^_2_	*N* = 239 *C* = 495 *a* = 0.1991		

**Table 2:** **Precision and recall of our approach evaluated on the 12 activities from in CAD60 and comparison with other algorithms**.

**Algorithm**	**Precision (%)**	**Recall (%)**	***F*-score (%)**
Sung et al., 2012	67.9	55.5	61.1
Ni et al., 2013	75.9	69.5	72.1
Koppula et al., 2013	80.8	71.4	75.8
Gupta et al., 2013	78.1	75.4	76.7
Gaglio et al., 2014	77.3	76.7	77
Zhang and Tian, 2012	86	84	85
Zhu et al., 2014	93.2	84.6	88.7
**Our approach**	**91.9**	**90.2**	**91**
Faria et al., 2014	91.1	91.9	91.5
Shan and Akella, 2014	93.8	94.5	94.1

Bold values indicate the classification results for our algorithm.

### 4.3. Evaluation

#### 4.3.1. Full-body actions

**Figure 6:**
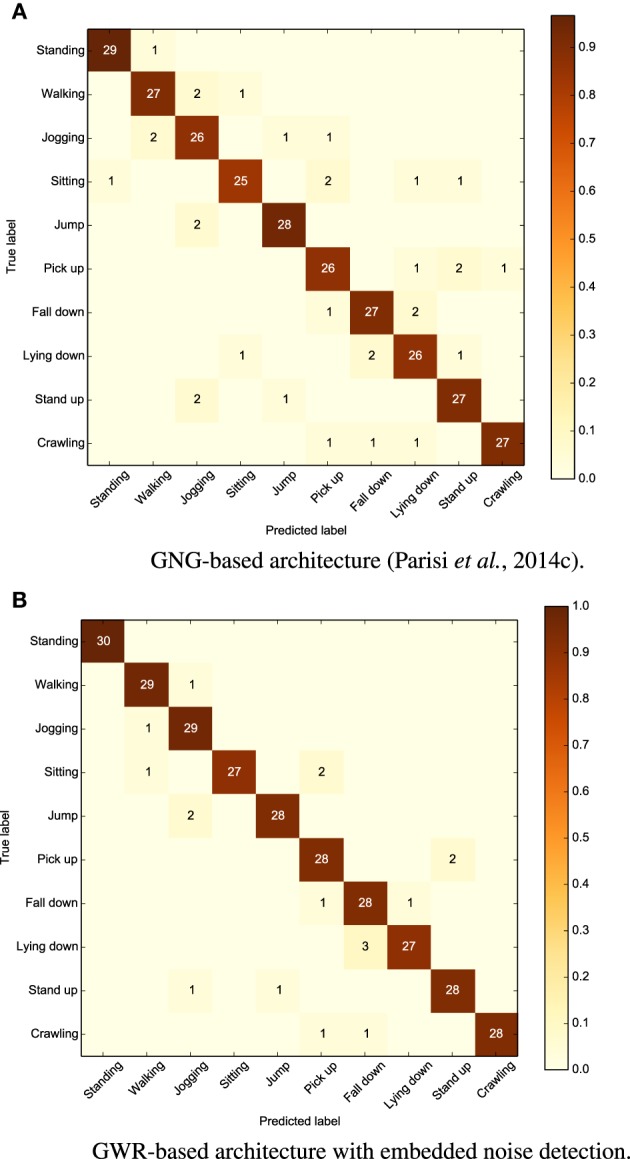
**Confusion matrices for our dataset of 10 actions showing better results for our GWR-based architecture (average accuracy 94%) compared to our previous GNG-based approach (89%)**.

Similar to previously reported results (Parisi et al., 2014c), we evaluated the system on 30 sequences of 10s for each periodic action and 30 repetitions for each goal-oriented action. Experiments showed that our new approach outperforms the previous one with an average accuracy rate of 94% (5% higher the than GNG-based architecture using an extra network for noise detection, and 18% higher than the same architecture without noise detection). We show the confusion matrix for both the approaches in Figure 6 (with each row of the matrix being an instance of the actual actions and each column an instance of the predicted actions). We can observe from the matrices that all the actions are slightly classified more accurately with respect to Parisi et al. (2014c). The most misclassified actions are “sitting” and “laying down.” In the first case, the action was confused with “walking” and “pick up.” This misclassification was mostly caused by skeleton tracking errors, i.e., when sitting down, the self-occlusion of joints may compromise the estimation of the overall body pose. The action “laying down” was, instead, misclassified as “fall down.” This is likely caused by the horizontal body poses shared between the two actions, despite the contribution of motion to disambiguate actions with similar poses.

#### 4.3.2. CAD60

For our evaluation on the CAD-60 dataset, we adopted a similar scheme as the one reported by Sung et al. (2012) using all the 12 activities plus a random action with *new person* strategy, i.e., the first 3 subjects for training and the remaining for test purposes. In Table 3, we show a comparison of our results with the state of the art on the CAD-60 dataset with precision and recall as evaluation metrics, and ranked by the *F*_1_-score computed as:

(14) $$F_{1}=2\cdot \frac{Precision\cdot Recall}{Precision+Recall}\;.$$

**Table 3:** **Precision, recall, and** ***F*****-score of our approach on the five environments of the CAD-60 dataset**.

**Location**	**Activity**	**Precision (%)**	**Recall (%)**	***F*-score (%)**
Office	Talking on the phone	94.1	92.8	93.4
	Drinking water	92.9	91.5	92.2
	Working on computer	94.3	93.9	94.1
	Writing on whiteboard	95.7	94.0	94.8
	Average	94.3	93.1	93.7
Kitchen	Drinking water	93.2	91.4	92.3
	Cooking (chopping)	86.4	86.7	86.5
	Cooking (stirring)	88.2	86.2	87.2
	Opening pill container	90.8	84.6	87.6
	Average	89.7	87.2	88.4
Bedroom	Talking on the phone	93.7	91.9	92.8
	Drinking water	90.9	90.3	90.6
	Opening pill container	90.8	90.1	90.4
	Average	91.8	91.7	91.7
Bathroom	Wearing contact lens	91.2	87.0	89.1
	Brushing teeth	90.6	88.0	89.3
	Rinsing mouth	87.9	85.8	86.8
	Average	89.9	86.9	88.4
Living room	Talking on the phone	94.8	92.1	93.4
	Drinking water	91.7	90.8	91.2
	Relaxing on couch	93.9	91.7	92.8
	Talking on couch	94.7	93.2	93.9
	Average	93.8	92.0	92.9

We obtained 91.9% precision, 90.2% recall, and 91% *F*-score, indicating that our model exhibits a good positive predictive value and very satisfactory sensitivity to classified actions. Precision and recall for each action and environment are shown in Table 3. To be noted is that we separated the actions into 5 different environments for a consistent and more informative comparison with other approaches using the same dataset, whereas the specific properties of the environments were not known to the model and had no effect on the segmentation of the skeleton joints, therefore not influencing the classification process.

The best state-of-the-art result has 93.8% precision, 94.5% recall, and 94.1% *F*-score (Shan and Akella, 2014). In their work, the authors identified a number of key poses prior to learning from which they compute spatio-temporal action templates, which makes this approach highly data-dependent. Each action must be segmented into atomic action templates composed of a set of *n* key poses, where *n* depends on the action's duration and complexity. Furthermore, experiments with low-latency (close to real-time) classification have not been reported. The second approach with slightly better results than ours is the work by Faria et al. (2014) with 93.2% precision, 91.9% recall, and 91.5% *F*-score. In their work, the authors used a dynamic Bayesian Mixture Model to classify motion relations between body poses. However, they used the raw depth images to estimate their own skeleton model (and did not use the one provided by the CAD-60 benchmark dataset). Therefore, differences in the tracked skeleton may exist that hinder a quantitative comparison with our classification method.

## 5. Discussion

### 5.1. Summary

In this paper, we presented a neurobiologically-motivated architecture that learns to recognize actions from depth map video sequences. The proposed approach relies on three assumptions that are consistent with evidence on neural mechanisms for action discrimination: (1) pose and motion action features are processed in two distinct pathways, respectively the ventral and the dorsal stream, and then action cues are integrated to provide a joint percept (Perrett et al., 1982; Vangeneugden et al., 2009); (2) hierarchies within each pathway process features with increasing complexity (Giese and Poggio, 2003); and (3) visual information is arranged according to input-driven self-organization (von der Malsburg, 1973; Kohonen, 1993; Miikkulainen et al., 2005). Our neural architecture consists of a two-pathway hierarchy of GWR networks that process pose-motion features in parallel and subsequently integrate action cues to provide movement dynamics in the joint feature space. Hierarchical learning was carried out using prototype trajectories composed of neuron activation patterns. The learning mechanism of the network allows to attenuate noise and detect noisy novel samples during on-line classification. For classification purposes, we extended the GWR implementation with two labeling functions. The evaluation of our approach has shown that our architecture outperforms previous results on action recognition for a dataset of 10 full-body actions, and that we achieved comparable results with the state of the art for a publicly available action benchmark.

Features of action sequences were extracted from depth map videos. The use of depth sensors has received increasing attention by action recognition researchers along with the integration of such technology with mobile robot platforms and humanoids (e.g., see Fanello et al., 2013; Parisi and Wermter, 2015). This is due to the fact that devices such as Kinect and Xtion represent low-cost sensors for the efficient segmentation of human motion robust to light changes in indoor environments. These factors play an important role in the development of a robust artificial system for the recognition of actions in real-world scenarios, e.g., detection of fall events in a domestic environment (Rougier et al., 2011; Mastorakis and Makris, 2012; Parisi and Wermter, 2015). Previous research has shown that the movement of a depth sensor, e.g., when mounted on a mobile robot, introduces a greater number of noisy observations that may impair the effective detection of action events (Parisi and Wermter, 2013). Therefore, artificial systems operating in natural environments should address the tolerance of noise to cope with sensor errors and occluded persons. The use of a self-organizing GWR allows to learn an incremental number of training actions and embed the mitigation of noisy samples into the learning mechanism. With this scheme, outliers in the training set do not propagate along the hierarchy during the training, and can automatically be detected during live classification (further details are discussed in Section 5.2).

### 5.2. Analogies with biological findings

The GWR networks (Marsland et al., 2002) have the ability to dynamically change their topological structure through competitive Hebbian learning (Martinetz, 1993) to incrementally match the distribution of the data in input space, thereby mimicking input-driven synaptic plasticity (Pascual-Leone et al., 2011) exhibited by some areas of the visual cortex (Hubel and Wiesel, 1962, 1977; Miikkulainen et al., 2005). Furthermore, this learning mechanism creates new neurons taking into account how well trained existing neurons are. This is achieved through a simplified model of the habituation process (Thompson and Spencer, 1966) and the benefits are twofold. First, it allows the convergence of the network in the sense that well-trained neurons will stop being updated. Second, the network responds quickly to changes in the distribution of the input. In this context, the insertion threshold has a strong influence on the number of neurons that will be created to match dynamic input fluctuations.

In our implementation of the GWR algorithm, we used the Euclidean distance as a metric to compute the distance of prototype neurons and neuron trajectories from the current input. Giese et al. (2008) investigated perceptual representations of full-body motion finding motion patterns that reside in perceptual spaces with well-defined metric properties. They conducted experiments with 2D and 3D joints of prototype trajectories with results implying that perceptual representations of complex motion patterns closely reflect the metric of movements in the physical world. Although more precise neural mechanisms that implement distance computation remain to be explored, we can therefore assume that the Euclidean distance is an adequate metric to compare articulated movement patterns.

For the processing of actions, we rely on the extraction of a simplified 3D skeleton model from which we estimate significant action properties, such as pose and motion, while maintaining a low-dimensional feature space. The skeleton model estimated by OpenNI, although not anatomically faithful, provides a convenient representation from which it is possible to extrapolate actor-independent action dynamics. The use of such models is in line with biological evidence demonstrating that human observers are very proficient at recognizing and learning complex motion underlying a skeleton structure (Jastorff et al., 2006; Hiris, 2007). These studies show that the presence of a holistic structure improves the learning speed and accuracy of action patterns, also for non-biologically relevant motion such as artificial complex motion patterns. This model may be susceptible to sensor noise and situations of partial occlusion and self-occlusion (e.g., caused by body rotation) for which body joint values may be noisy or missing. Although it may be desirable to implement invariance transformations (e.g., Sofatzis et al., 2014) or remove sensor noise (Parisi and Wermter, 2013), these limitations are not in contrast with biological evidence demonstrating that the recognition of complex motion is strongly view-dependent. Psychophysical studies showed that action recognition is impaired by biological motion stimuli being upside-down or rotated with respect to the image plane (Sumi, 1984; Pavlova and Sokolov, 2000). Furthermore, it has been found that learned visual representations seem to be highly orientation-dependent, i.e., discrimination performance increased only when the test patterns presented the same orientation as in the training (Jastorff et al., 2006). Therefore, view-dependence in recognition of complex motion is consistent with the idea that recognition is based on the matching of learned two-dimensional patterns, whereas view-independence may be achieved by means of 3D internal models (Hogg, 1983).

Our recognition scheme for action sequences is in line with a number of studies demonstrating that action discrimination is selective to temporal order (Bertenthal and Pinto, 1993; Giese and Poggio, 2003; Jastorff et al., 2006). Therefore, this task may involve learning mechanisms able to extrapolate spatio-temporal dependencies of sequences. Recurrent versions of self-organizing networks have been extensively investigated that extend the feed-forward learning mechanism with context neurons for referring to past activations, thereby allowing the processing of sequences and structured data (e.g., see Strickert and Hammer, 2005 for a recursive SOM model). Although the original implementation of the GWR processes real-valued vectors only in the spatial domain, it may be easily extended for processing sequences in a similar fashion. For instance, Andreakis et al. (2009) devised a recursive GNG network with a context layer to learn spatio-temporal patterns. However, consistently with evidence of a hierarchical architecture of the visual system (Giese and Poggio, 2003), we opted for a feed-forward architecture that exhibits progressively time-selective levels of representations. In this setting, action recognition is modulated by temporal order resulting from lateral connections that form activation trajectories between prototype neurons. Trajectories were generated with serialized concatenations of a fixed number of samples in a temporal sliding window fashion, in our specific case empirically set to trajectories of 3 neuron activations for each visual cue. This scheme is in accordance with neurophysiological evidence that actions are represented by sequences of integrated poses over fixed windows of around 120 ms (Singer et al., 2010). A series of well-established computational models have been proposed that implement a feed-forward architecture for processing action features with increasing complexity (Giese and Poggio, 2003; Lange et al., 2006; Tan et al., 2013).

### 5.3. Future work

In this work, we focused on a feed-forward mechanism for learning human actions represented with pose-motion features. However, a number of studies have demonstrated that biological motion recognition is also strongly modulated by higher level cognitive representations, such as top-down influences (Bülthoff et al., 1998; Thornton et al., 2002), and representations of biomechanically plausible motion (Shiffrar and Freyd, 1990). These aspects were not considered in this paper and are part of future work.

An additional future work direction is to investigate the interplay of pose-motion cues and recognition strategies when one of the two stimuli is suppressed. At its current state, our system requires that both the pose and motion samples are available for parallel processing and integration. However, studies have shown that observers can shift between pose and motion-based strategies, depending on the available cue (Tyler et al., 2011). In other words, suppressing one of the cues does not fully impair action perception. In line with this assumption, we could extend our neural architecture with interlateral connections so that neurons from distinct pathways can co-activate in the presence of single-cue input. With our implementation, this mechanism would require neurons in GWR to be equipped with symmetric, inter-network references that link prototype neurons between the *G*^*P*^ and *G*^*M*^ populations, and enable the computing of activation trajectories in both pathways when only neurons from one pathway are activated. In this setting, the dynamics of learning and cue integration are to be investigated.

Finally, the reported results motivate the embedding of our learning system into mobile robot platforms to conduct further evaluations in more complex scenarios, where the robust recognition of actions plays a key role. For instance, the visual detection of dangerous events for assistive robotics such as fall events (Parisi and Wermter, 2013, 2015), and the recognition of actions with learning robots in HRI scenarios (Soltoggio et al., 2013a,b; Barros et al., 2014; Parisi et al., 2014a,b).

### Conflict of interest statement

The authors declare that the research was conducted in the absence of any commercial or financial relationships that could be construed as a potential conflict of interest.

## Footnotes

^1^OpenNI SDK. http://openni.ru/openni-sdk/.

## References

[B1] Andreakis N. Hoyningen-Huene N. v. Beetz M. (2009). Incremental unsupervised time series analysis using merge growing neural gas, in Advances in Self-Organizing Maps, eds Principe J. C. Miikkulainen R. (St. Augustine, FL: Springer), 10–18.

[B2] Bülthoff I. Bülthoff H. Sinha P. (1998). Top-down influences on stereoscopic depth perception. Nat. Neurosci. 1, 254–257. 10.1038/699 10195152

[B3] Barros P. Parisi G. I. Jirak D. Wermter S. (2014). Real-time gesture recognition using a humanoid robot with a deep neural Architecture, in 2014 14th IEEE-RAS International Conference on Humanoid Robots (Humanoids) (Madrid: IEEE-RAS), 83–88.

[B4] Beauchamp M. S. Lee K. E. Haxby J. V. Martin A. (2003) FMRI responses to video and point-light displays of moving humans and manipulable objects. J. Cogn. Neurosci. 15, 991–1001. 10.1162/089892903770007380 14614810

[B5] Bertenthal B. I. Pinto J. (1993). Complementary processes in the perception and production of human movement, in Dynamic Approaches to Development 2, eds Thelen E. Smith L. (Cambridge: MIT Press), 209–239.

[B6] Beyer O. Cimiano P. (2011) Online labelling strategies for growing neural gas, in IDEAL11, eds Yin H. Wang W. Rayward-Smith V. (Norwich: Springer Berlin Heidelberg), 76–83.

[B7] Blake R. Shiffrar M. (2007). Perception of human motion. Annu. Rev. Psychol. 58, 47–73. 10.1146/annurev.psych.57.102904.190152 16903802

[B8] Ellis C. Masood S. Z. Tappen M. F. LaViola J. J. Jr. Sukthankar R. (2013). Exploring the trade-off between accuracy and observational latency in action recognition. Int. J. Comput. Vis. 101, 420–436. 10.1007/s11263-012-0550-7

[B9] Fanello S. R. Gori I. Metta G. Odone F. (2013) Keep it simple and sparse: real-time action recognition. J. Mach. Learn. Res. 14, 2617–2640.

[B10] Faria D. R. Premebida C. Nunes U. (2014). A probabilistic approach for human everyday activities recognition using body motion from RGB-D images, in 2014 RO-MAN: the 23rd IEEE International Symposium Robot and Human Interactive Communication (Edinburgh: IEEE).

[B11] Fritzke B. (1995). A growing neural gas network learns topologies, in Advances in Neural Information Processing Systems, Vol. 7, eds Tesauro G. Touretzky D. S. Leen T. K. (Cambridge, MA: MIT Press), 625–632.

[B12] Gaglio S. Lo Re M. Morana M. (2014) Human activity recognition process using 3-D posture data. IEEE Trans. Hum. Mach. Syst. 99, 1–12. 10.1109/THMS.2014.2377111

[B13] Garcia J. O. Grossman E. D. (2008). Necessary but not sufficient: motion perception is required for perceiving biological motion. Vis. Res. 48, 1144–1149. 10.1016/j.visres.2008.01.027 18346774

[B14] Giese M. A. Poggio T. (2003). Neural mechanisms for the recognition of biological movements. Nat. Rev. Neurosci. 4, 179–192. 10.1038/nrn1057 12612631

[B15] Giese M. A. Thornton I. Edelman S. (2008). Metrics of the perception of body movement. J. Vis. 8, 1–18. 10.1167/8.9.13 18831649

[B16] Gupta R. Chia A. Y.-S. Rajan D. (2013). Human activities recognition using depth images, in ACM International Conference on Multimedia (New York, NY: ACM), 283–292.

[B17] Han J. Shao L. Xu D. Shotton J. (2013) Enhanced computer vision with microsoft kinect sensor: a review. IEEE Trans. Cybern. 43, 1318–1334. 10.1109/TCYB.2013.2265378 23807480

[B18] Hebb D. O. (1949). The Organization of Behavior. New York, NY: Wiley.

[B19] Hiris E. (2007) Detection of biological and nonbiological motion. J. Vis. 7, 1–16. 10.1167/7.12.4 17997646

[B20] Hogg D. (1983). Model-based vision: a program to see a walking person. Image Vis. Comp. 1, 5–19. 10.1016/0262-8856(83)90003-3

[B21] Hubel D. H. Wiesel T. N. (1962). Receptive fields, binocular interaction and functional architecture in the cat's visual cortex. J. Physiol. 160, 106–154. 10.1113/jphysiol.1962.sp006837 14449617 PMC1359523

[B22] Hubel D. H. Wiesel T. N. (1977). Ferrier lecture. Functional architecture of macaque monkey visual cortex. Proc. R. Soc. Lond. B Biol. Sci. 198, 1–59. 10.1098/rspb.1977.0085 20635

[B23] Jastorff J. Kourtzi Z. Giese M. A. (2006). Learning to discriminate complex movements: biological versus artificial trajectories. J. Vis. 6, 791–804. 10.1167/6.8.3 16895459

[B24] Jiang Z. Lin Z. Davis L. S. (2012). Recognizing human actions by learning and matching shape-motion prototype trees. IEEE Trans. Pattern Anal. Mach. Intell. 34, 533–547. 10.1109/TPAMI.2011.147 21788666

[B25] Kachouie R. Sedighadeli S. Khosla R. Chu M-T. (2014). Socially assistive robots in elderly care: a mixed-method systematic literature review. Int. J. Hum. Comput. Interact. 30, 369–393. 10.1080/10447318.2013.873278

[B26] Kohonen T. (1993). Self-Organization and Associative Memory, 3rd Edn. Berlin: Springer.

[B27] Kohonen T. (1995). Self-organizing maps, in Series in Information Science 30, eds Huang T. S. Kohonen T. Schroeder M. R. (Heidelberg: Springer), 502.

[B28] Koppula H. S. Gupta R. Saxena A. (2013). Learning human activities and object affordances from RGB-D videos. Int. J. Robot. Res. 32, 951–970. 10.1177/0278364913478446

[B29] Lange J. Lappe M. (2006). A model of biological motion perception from configural form cues. J. Neurosci. 26, 2894–2906. 10.1523/JNEUROSCI.4915-05.2006 16540566 PMC6673973

[B30] Layher G. Giese M. A. Neumann H. (2012). Learning representations for animated motion sequence and implied motion recognition, in ICANN12 (Helderberg: Springer), 427–434.

[B31] Marsland S. Shapiro J. Nehmzow U. (2002). A self-organising network that grows when required. Neural Netw. 15, 1041–1058. 10.1016/S0893-6080(02)00078-3 12416693

[B32] Martinetz T. Schluten K. (1991). A “neural-gas” network learns topologies, in Artificial Neural Networks, eds Kohonen T. Makisara K. Simula O. Kangas J. (North Holland: Elsevier Science Publishers B.V.), 397–402.

[B33] Martinetz T. (1993). Competitive Hebbian learning rule forms perfectly topology preserving maps, in ICANN93 (Helderberg: Springer), 427–434.

[B34] Mastorakis G. Makris D. (2012). Fall detection system using Kinect's infrared sensor. J. Real Time Image Process 9, 635–646.

[B35] Meltzoff A. N. Moore M. K. (1977). Imitation of facial and manual gestures by human neonates. Science 198, 74–78. 10.1126/science.897687 897687

[B36] Miikkulainen R. Bednar J. A. Choe Y. Sirosh J. (2005) Computational Maps in the Visual Cortex. New York, NY: Springer.

[B37] Neri P. Concetta Morrone M. Burr D. C. (1998). Seeing biological motion. Nature 395, 894–896. 10.1038/27661 9804421

[B38] Ni B. Pei Y. Moulin P. Yan S. (2013). Multilevel depth and image fusion for human activity detection. IEEE Trans. Cybern. 43, 1383–1394. 10.1109/TCYB.2013.2276433 23996589

[B39] Oram M. W. Perrett D. I. (1996). Integration of form and motion in the anterior superior temporal polysensory area (STPa) of the macaque monkey. J. Neurophysiol. 76, 109–129. 8836213 10.1152/jn.1996.76.1.109

[B40] Papadopoulos G. Th. Axenopoulos A. Daras P. (2014). Real-time skeleton-tracking-based human action recognition using kinect data, in MultiMedia Modeling '14 eds Gurrin C. Hopfgartner F. Hurst W. Johansen H. Lee H. O'Connor N. (Dublin: Springer International Publishing), 473–483.

[B41] Parisi G. I. Wermter S. (2013). Hierarchical SOM-based detection of novel behavior for 3D human tracking, in The 2013 International Joint Conference on Neural Networks (Dallas, TX: IEEE), 1380–1387.

[B42] Parisi G. I. Barros P. Wermter S. (2014a). FINGeR: framework for interactive neural-based gesture recognition, in ESANN14 443–447.

[B43] Parisi G. I. Jirak D. Wermter S. (2014b). HandSOM - neural clustering of hand motion for gesture recognition in real time, in Proceedings of the IEEE International Symposium on Robot and Human Interactive Communication (RO-MAN '14) (Edinburgh: IEEE), 981–986.

[B44] Parisi G. I. Weber C. Wermter S. (2014c). Human action recognition with hierarchical growing neural gas learning, in ICANN14 (Helderberg: Springer), 89–96.

[B45] Parisi G. I. Wermter S. (2015). Neurocognitive assistive robot for robust fall detection. Smart Environ.

[B46] Pascual-Leone A. Freitas C. Oberman L. Horvath J. C. Halko M. Eldaief M.. (2011). Characterizing brain cortical plasticity and network dynamics across the age-span in health and disease with TMS-EEG and TMS-fMRI. Brain Topogr. 24, 302–315. 10.1007/s10548-011-0196-8 21842407 PMC3374641

[B47] Pavlova M. Sokolov S. (2000). Orientation specificity in biological motion perception. Percept. Psychophys. 62, 889–899. 10.3758/BF03212075 10997036

[B48] Pavlova M. Krageloh-Mann I. Sokolov A. Birbaumer N. (2001). Recognition of point-light biological motion displays by young children. Perception 30, 925–933. 10.1068/p3157 11578078

[B49] Perrett D. I. Rolls E. T. Caan W. (1982). Visual neurons responsive to faces in the monkey temporal cortex. Exp. Brain Res. 47, 329–342. 10.1007/BF00239352 7128705

[B50] Rougier C. Auvinet E. Rousseau J. Mignotte M. Meunier J. (2011). Fall detection from depth map video sequences, in Toward Useful Services for Elderly and People with Disabilities, eds Abdulrazak B. Giroux S. Bouchard B. Pigot H. Mokhtari M. (Montreal, QC: Springer Berlin - Heidelberg), 121–128.

[B51] Shan J. Akella S. (2014). 3D Human action segmentation and recognition using pose kinetic energy, in Workshop on Advanced Robotics and its Social Impacts (ARSO14) (Evanston, IL: IEEE), 69–75.

[B52] Shiffrar M. Freyd J. J. (1990). Apparent motion of the human body. Psychol. Sci. 1, 257–264. 10.1111/j.1467-9280.1990.tb00210.x

[B53] Singer J. M. Sheinberg D. L. (2010). Temporal cortex neurons encode articulated actions as slow sequences of integrated poses. J. Neurosci. 30, 3133–3145. 10.1523/JNEUROSCI.3211-09.2010 20181610 PMC3669686

[B54] Sofatzis R. J. Afshar S. Hamilton T. J. (2014). Rotationally invariant vision recognition with neuromorphic transformation and learning networks, in 2014 IEEE International Symposium on Circuits and Systems (Melbourne, VIC: IEEE), 469–472.

[B55] Soltoggio A. Lemme A. Reinhart F. Steil J. (2013a). Rare neural correlations implement robotic conditioning with delayed rewards and disturbances. Front. Neurorobot. 7:6. 10.3389/fnbot.2013.00006 23565092 PMC3613617

[B56] Soltoggio A. Reinhart F. Lemme A. Steil J. (2013b). Learning the rules of a game: neural conditioning in human-robot interaction with delayed rewards, in IEEE International Conference of Development and Learning and on Epigenetic Robotics (Osaka).

[B57] Stanley J. C. (1976). Computer simulation of a model of habituation. Nature 261, 146–148. 10.1038/261146a0 179015

[B58] Strickert M. Hammer B. (2005). Merge SOM for temporal data. Neurocomputing 64, 39–71. 10.1016/j.neucom.2004.11.01418815099

[B59] Suarez J. Murphy R. (2012). Hand gesture recognition with depth images: a review, in RO-MAN14 (Edinburgh, UK: IEEE), 411–417.

[B60] Sumi S. (1984). Upside-down presentation of the Johansson moving light-spot pattern. Perception 13, 283–302. 10.1068/p130283 6514513

[B61] Sung J. Ponce C. Selman B. Saxena A. (2012). Unstructured human activity detection from RGBD images, in 2012 IEEE International Conference on Robotics and Automation (Saint Paul, MN: IEEE), 842–849.

[B62] Tan C. Singer J. M. Serre T. Sheinberg D. Poggio T. A. (2013). Neural representation of action sequences: how far can a simple snippet-matching model take us? in Advances in Neural Information Processing Systems, NIPS13 (Lake Tahoe, CA: Harrahs and Harveys), 1–9.

[B63] Thirkettle M. Benton C. P. Scott-Samuel N. E. (2009). Contributions of form, motion and task to biological motion perception. J. Vis. 9, 1–11. 10.1167/9.3.28 19757967

[B64] Thompson R. F. Spencer W. A. (1966). Habituation: a model phenomenon for the study of neuronal substrates of behavior. Psychol. Rev. 73, 16–43. 10.1037/h0022681 5324565

[B65] Thornton I. M. Rensink R. A. Shiffrar M. (2002). Active versus passive processing of biological motion. Perception 31, 837–853. 10.1068/p3072 12206531

[B66] Troje N. F. (2002). Decomposing biological motion: a framework for analysis and synthesis of human gait patterns. J. Vis. 2, 371–387. 10.1167/2.5.2 12678652

[B67] Tyler S. C. Grossman E. D. (2011). Feature-based attention promotes biological motion recognition. J. Vis. 11, 1–6. 10.1167/11.10.11 21926183

[B68] Vangeneugden J. Pollick F. Vogels R. (2009). Functional differentiation of macaque visual temporal cortical neurons using a parametric action space. Cereb. Cortex 19, 593–611. 10.1093/cercor/bhn109 18632741

[B69] von der Malsburg C. (1973). Self-organization of orientation sensitive cells in the striate cortex. Kybernetik 14, 85–100. 10.1007/BF00288907 4786750

[B70] Weinland D. Ronfard R. Boyer R. (2011). A survey of vision-based methods for action representation, segmentation and recognition. Comput. Vis. Image Underst. 115, 224–241. 10.1016/j.cviu.2010.10.002

[B71] Xu R. Agarwal P. Kumar S. Krovi V. N. Corso J. J. (2012). Combining skeletal pose with local motion for human activity recognition, in Articulated Motion and Deformable Objects (LNCS 7378), eds Perales F. J. Fisher R. B. Moeslund T. B. (Mallorca: Springer Berlin Heidelberg) 114–123.

[B72] Yanik P. M. Manganelli J. Merino J. Threatt A. L. Brooks J. O. Evan Green K. (2014) A gesture learning interface for simulated robot path shaping with a human teacher. IEEE Trans. Hum. Mach. Syst. 44, 44–51. 10.1109/TSMC.2013.2291714

[B73] Zhang C. Tian Y. (2012). RGB-D camera-based daily living activity recognition. J. Comput. Vis. Image Process. 2, 1–7.

[B74] Zhu Y. Chen W. Guo G. (2014). Evaluating spatiotemporal interest point features for depth-based action recognition. Image Vis. Comput. 32, 453–464. 10.1016/j.imavis.2014.04.00524577192

